# Assessing the Quality of Life of Pregnant Women in Romania: Socioeconomic, Health, and Obstetric Factors and the Validation of the WHOQOL-BREF Instrument

**DOI:** 10.3390/nursrep15030078

**Published:** 2025-02-26

**Authors:** Mihaela Corina Radu, Sebastian Mihai Armean, Laura Ioana Chivu, Justin Aurelian, Cosmin Medar, Loredana Sabina Cornelia Manolescu

**Affiliations:** 1 Department of Microbiology, Parasitology and Virology, Faculty of Midwives and Nursing, “Carol Davila” University of Medicine and Pharmacy, 020021 Bucharest, Romania; loredana.manolescu@umfcd.ro; 2 Emergency Hospital County, Str. Mihai Bravu No. 106, 100409 Ploieşti, Romania; 3 Department of Nursing, Faculty of Midwifery and Nursing, “Carol Davila” University of Medicine and Pharmacy, 020021 Bucharest, Romania; 4 Department of Pharmacology, Toxicology and Clinical Pharmacology, Faculty of Medicine, “Iuliu Hateganu” University of Medicine and Pharmacy, Str. Victor Babes, No. 8, 400347 Cluj Napoca, Romania; sebastian.armean@umfcluj.ro; 5 Department of Pathophysiology, Faculty of Midwifery and Nursing, “Carol Davila” University of Medicine and Pharmacy, 050474 Bucharest, Romania; 6 “Prof Dr. Th. Burghele” Clinical Hospital, 061344 Bucharest, Romania; 7 Department of Clinical Laboratory of Radiology and Medical Imaging, Faculty of Midwives and Nursing, “Carol Davila” University of Medicine and Pharmacy, 020021 Bucharest, Romania; cosmin.medar@umfcd.ro

**Keywords:** quality of life, pregnant women, socioeconomic factors, health factors, obstetric factors, WHOQOL-BREF, psychometric validation, maternal health, pregnancy assessment

## Abstract

Pregnancy is a transformative stage in a woman’s life, marked by significant physical, emotional, and social changes. This study had three main **objectives:** (1) to assess the quality of life (QoL) of pregnant women in Romania, (2) to identify the sociodemographic, health, and obstetric factors influencing their QoL and (3) to examine the psychometric properties of the WHOQOL-BREF questionnaire within the Romanian context, determining its effectiveness in evaluating QoL during pregnancy. **Methods:** A cross-sectional analytical survey was conducted between January and July 2023 among pregnant women in Romania, targeting a geographically diverse sample from urban and rural areas. Eligible participants were Romanian citizens aged 18 or older. Data were collected through an online self-administered questionnaire using Google Forms, with informed consent obtained electronically. The survey included demographic, obstetric, and health-related variables alongside the WHOQOL-BREF tool, which evaluates QoL across four dimensions: Physical, Psychological, Social Relationships, and Environment. Statistical analysis involved confirmatory factor analysis, reliability testing (Cronbach’s α and McDonald’s ω), and comparisons using Welch’s t-tests and ANOVA. **Results:** A total of 1550 valid responses were analyzed. The WHOQOL-BREF demonstrated excellent internal consistency (Cronbach’s α > 0.9 across all dimensions). Women with higher education and stable employment reported significantly higher QoL scores in physical and psychological dimensions. No significant differences were found based on pregnancy trimester, previous births, or participation in prenatal classes, although trends suggested slight advantages for participants in prenatal education. Women delivering in private hospitals or non-hospital settings reported better psychological and physical QoL than those delivering in public hospitals. Support from partners and urban residency positively influenced perceived QoL. **Conclusions:** The WHOQOL-BREF is a reliable tool for assessing QoL in pregnant women in Romania. The study highlights the role of education, employment, and delivery location in influencing QoL, emphasizing the need for targeted support for vulnerable groups during pregnancy.

## 1. Introduction

Pregnancy is a unique stage in a woman’s life, characterized by significant physical, emotional, and social changes. While for many women it represents a moment of joy and personal fulfillment, pregnancy can also bring challenges that profoundly affect quality of life. This period involves physiological changes such as weight gain, hormonal fluctuations, and physical discomfort, as well as psychological factors like anxiety, stress, or fear of childbirth [1,2,3].

Romania is part of the group of post-communist countries that have experienced a significant decline in fertility rates, a phenomenon closely linked to the major economic, social, and political transformations that occurred after the fall of the communist regime. This decrease in fertility coincided with massive emigration, especially towards Western European countries, which has amplified the country’s demographic decline over the past three decades.

Today, in Romania, more and more women are postponing the decision to become mothers, a complex phenomenon influenced by a range of economic, social, and cultural factors. Financial instability is one of the most important factors, given the high costs associated with raising a child, difficulties in accessing housing, and job insecurity. Additionally, career prioritization plays an essential role, as more women aim to strengthen their professional positions before taking on the responsibility of motherhood. The fast pace of modern life and societal pressures for professional and educational performance further contribute to delaying the decision to have children, especially among women with higher education and stable careers [4,5,6,7,8].

These trends not only affect the physical health of pregnant women but also have a considerable psychological impact, as pregnancy at older ages is associated with additional risks and complications that can diminish quality of life [8,9,10].

In Romania, where access to healthcare services, social support, and reproductive health education varies significantly depending on region and environment, the impact of pregnancy on quality of life can differ from global contexts [2,3]. Additionally, limited access to prenatal education and quality care may amplify these challenges for many pregnant women. At the same time, family, community, and professional support can positively influence women’s perceptions and experiences during pregnancy [8,9,11,12].

From a psychological perspective, maternal identity formation begins even before birth, involving the creation of an idealized self-image as a mother and about the child. This process fosters a sense of responsibility, which leads to changes in family and social dynamics [13,14].

In the obstetric context, unplanned pregnancies are associated with gestational depression, financial difficulties, and negative impacts on personal and professional life. Furthermore, as pregnancy progresses, sleep quality deteriorates due to muscle pain, breathing difficulties, and heartburn [15,16,17].

The experience of pregnancy is also influenced by personal circumstances such as marital status, socioeconomic conditions, and cultural beliefs. Sociodemographic factors such as advanced maternal age, low education levels, common pregnancy symptoms, and the quality of prenatal care are significant determinants of the impact of pregnancy on quality of life [2,7,8,18].

Fear of childbirth negatively affects women’s health during and after pregnancy and can lead to adverse perinatal outcomes and psychological consequences such as prolonged and complicated labor, emotional imbalance (e.g., anxiety or irritability), difficulties in the mother-child relationship, increased risk of postpartum depression, and maternal post-traumatic stress disorder. Moreover, fear of childbirth contributes to the rise in unnecessary cesarean sections without medical indication and the high number of medicalized births [2,7,8,18,19].

Research on the quality of life of pregnant women is essential to analyze life contexts and factors influencing quality of life during pregnancy, as it helps identify the most vulnerable groups requiring greater attention. Understanding the quality of life of pregnant women is crucial, as those with lower quality of life are at higher risk of complications during pregnancy. Such complications require treatments that impose additional stress [4,11,20].

Currently, several research studies and articles attempt to define the concept of quality of life by addressing its various aspects or domains [21,22,23,24]. The relationship between the normal physiological process of pregnancy and a woman’s quality of life during this period receives much less attention [25,26].

The World Health Organization Quality of Life Questionnaire (WHOQOL-BREF) is one of the most well-known generic tools for assessing quality of life (QoL) in both healthy and ill populations [27,28,29,30]. Over the past two decades, a WHOQOL-BREF manual has facilitated approximately 100 culturally adapted translations of this tool globally, completed by more than 60,000 adults in healthy and ill populations [31]. Validating measurement instruments, including WHOQOL-BREF, is an ongoing process, requiring accumulated evidence of validity to support the inferences and interpretations of the tool’s scores [32]. Research on the psychometric properties of WHOQOL-BREF generally indicates satisfactory validity and reliability of the scale [27,28,29,33,34].

In this study, we focus on assessing the psychometric properties of the Romanian version of WHOQOL-BREF. We aim to replicate previous research on these properties in a population of pregnant women and expand the research by testing measurement invariance by age, education level, ethnicity, residence, income, and marital status. Finally, we aim to provide updated normative data for a population of pregnant women in Romania.

Research conducted on a population of pregnant women is crucial for analyzing life contexts and factors influencing quality of life during pregnancy, as it helps identify the most vulnerable groups that need more attention. Thus, the purpose of this study was to analyze the quality of life and its association with demographic, socioeconomic, obstetric, and health conditions of pregnant women.

The aim of this study was to describe the perception of changes brought about by pregnancy, including their impact on quality of life, and to identify the variables that, from the perspective of pregnant women in Romania, significantly affect their quality of life. Additionally, the study aimed to examine the properties of the generic WHOQOL-BREF questionnaire and determine whether it is sufficiently sensitive to provide an optimal assessment of these women’s quality of life.

## 2. Materials and Methods

### 2.1. Study Design and Setting

This study targeted pregnant women from the general population of Romania, residing in various counties and the country’s capital, Bucharest. The inclusion criteria required participants to be pregnant, at least 18 years old, and hold Romanian citizenship.

### 2.2. Participants and Data Collection

Data were collected through a self-administered questionnaire, with informed consent obtained from each participating pregnant woman. The questionnaire was administered online via the cloud-based Google Forms platform and was available on the internet for seven months, between January and July 2023.

The study aimed to involve pregnant women from across Romania, covering a wide geographical area, including counties and the capital. To facilitate the participation of as many women as possible, modern recruitment methods were used, relying on the electronic distribution of an announcement through online platforms. These platforms provided quick and easy access to a broad audience, increasing the chances of obtaining a diverse and representative sample of the general population.

The announcement was designed to provide pregnant women with all necessary information about the study’s purpose and methodology, emphasizing the importance of research in the field of reproductive health and birth management in Romania. Upon accessing the link included in the announcement, interested women could read a clear and concise description of the study, focusing on the implemented safety and confidentiality measures. To participate, women had to provide informed consent, a crucial step in any research involving human participants. This consent was obtained electronically, through their choice to proceed with completing the questionnaire after acknowledging all study details. The informed consent process ensured not only adherence to research ethics but also the protection of participants’ rights and interests. This approach, using electronic means and a clear participation framework, allowed pregnant women to partake in the research comfortably, anonymously, and safely, without external pressure or influence, while ensuring an efficient flow of relevant data for the study’s objectives.

### 2.3. Instruments

The WHOQOL-BREF questionnaire was used to measure quality of life. This questionnaire is the most commonly utilized generic tool for assessing quality of life during pregnancy. The WHOQOL-BREF contains a total of 26 items grouped into four domains, as well as two separate questions evaluating overall quality of life (Q1) and satisfaction with health status (Q2). Results are expressed through four domain scores and the mean raw scores of the two separate items:

D1-Physical Health (7 items): Includes questions about mobility, daily activities, functional capacity, energy, pain, and sleep.

D2-Psychological Health (6 items): Covers self-image, negative thoughts, positive attitudes, self-esteem, mindset, learning ability, memory, concentration, religion, and mental state.

D3-Social Relationships (3 items): Focuses on personal relationships, social support, and sexual life.

D4-Environment (8 items): Encompasses aspects such as financial resources, safety, health and social services, living conditions, opportunities to acquire new skills, recreation, general environment (noise, air pollution, etc.), and transportation.

WHOQOL-BREF uses a five-point rating scale. It is evaluated as follows: 1 = very poor, 2 = slightly poor, 3 = neither good nor bad, 4 = fairly good, and 5 = very good. The higher the score, the better the quality of life, and in each domain, it is expressed as average values calculated according to the guidelines and instructions.

There is no total score for WHOQOL-BREF. If one item is missing, the mean of the remaining items in the domain can be used. If more than two items are missing from a domain, the domain score should not be calculated, except for Domain 3, where more than one missing item nullifies the calculation. Questionnaires with more than 20% missing items should also be excluded. The Romanian version of this scale was translated according to the WHO translation protocol [23].

### 2.4. Data Analysis

The following variables were selected as exposures:

Demographic Characteristics: Information on age, ethnicity, residence, income, marital status, and highest educational qualification completed.

Health Status Measures: Current pregnancy status and stage (trimester), with non-pregnancy being an exclusion criterion, previous births (viable births and type: vaginal delivery, cesarean section), frequency of consultations during the current pregnancy with a family physician and/or a specialist in public or private sectors, preferred birth location, and expectations regarding autonomy in decision-making during pregnancy and childbirth.

### 2.5. Statistical Analysis

For statistical analysis, JASP 0.18.3 R © JASP Team (2024) and R version 4.3.3 (Copyright © 2024, The R Foundation for Statistical Computing) were used. Specific R packages included EFA.dimensions, EFAtools, lavaan, semPlots, and gtsummary [35,36,37,38,39].

Data processing involved the COUNTIFS function in Microsoft Office Excel for filtering and sorting the initial database. Categorical variables related to socio-demographic characteristics and obstetric profiles of pregnant women were expressed using descriptive statistics and frequencies. Welch’s two-sided *t*-tests were employed for categorical variables with two categories, while ANOVA tests followed by post-hoc procedures were applied for variables with more than two categories. A *p*-value < 0.05 was adopted as the threshold for statistical significance.

The analysis consisted of the following steps:

A consistency analysis was performed for the total questionnaire and then separately for the four domains, followed by a confirmatory factor analysis for the proposed structure with four domains: Physical, Psychological, Social Relationships, and Environment.

Transformed scores on a 0–100 scale were calculated for each domain of the questionnaire.

### 2.6. Ethical Approval

The questionnaire was peer-reviewed and approved by the Ethics Committee of Dr. Constantin Andreoiu Emergency Hospital in Ploiești, Romania (41482/9 August 2022); all study procedures adhered to the ethical standards of the Declaration of Helsinki. Informed consent was mandatory.

## 3. Results

A total of 1567 individual responses were collected. Seventeen participants were excluded due to incomplete responses. After validating the test, 1550 participants remained. The analysis of the socio-demographic and obstetric profile of the pregnant women revealed that approximately half, 760 (49.03%), of the participants were aged between 18 and 29 years, and 1170 (75.48%) of them were married. Among the 1550 pregnant women, 1270 (81.93%) had higher education, 1240 (80%) had a regular job, and the vast majority, 1499 (96.70%), were of Romanian ethnicity. Two-thirds of the survey participants lived in an urban area, 1090 (70.32%), while 510 (32.90%) had attended childbirth education classes, and 1130 (72.90%) were in their third trimester of pregnancy. Additionally, 160 (10.32%) of the respondents did not wish to give birth in a hospital, and 20 (0.12%) reported having more than five previous births.

Consistency analysis for the entire questionnaire (Table 1):

The scale demonstrates excellent internal consistency, as evidenced by McDonald’s Omega and Cronbach’s Alpha both being well above 0.9. The average inter-item correlation indicates that the items on the scale are moderately correlated, which is typical for a well-constructed scale. The narrow confidence intervals for both reliability estimates and the average inter-item correlation suggest that these statistics are robust and reliable. In conclusion, the scale used in the study is highly reliable, and the items consistently measure the same underlying construct. This high level of reliability supports the validity of any conclusions drawn from the data collected using this scale.

Most items on this scale show moderate to high correlations, suggesting that the items are well-aligned and contribute positively to the internal consistency of the scale.

Items with higher correlations are the strongest predictors of the total score and are essential for assessing the scale’s reliability. The results indicate good consistency for the overall questionnaire. To analyze the given Frequentist Individual Item Reliability Statistics and the item-rest correlations for the dataset, we will look at the correlations for each item to understand their reliability within the corresponding dimensions (D1, D2, D3, and D4) (Table 2).

The items in Dimension 1 generally show moderate to high item-rest correlations, with I17 and I18 having the highest reliability indicators. Items I4 and I15 have relatively lower correlations but are still within acceptable ranges. Dimension 2 items also show moderate correlations. The lowest correlation is for I26, which indicates it might be less reliable within this dimension compared to others. The items in Dimension 3 display moderate reliability, with I21 showing the lowest correlation, suggesting it might not be as reliable as other items in this dimension. In conclusion, the items in Dimension 4 exhibit moderate to high correlations, suggesting overall good reliability.

Analysis for each domain (Table 3):

The results indicate good consistency for the Physical, Psychological, and Environment domains. The consistency of the Social domain was acceptable, considering the number of items, with the results being compatible with those obtained from the samples used by the authors for the questionnaire’s validation.

For the confirmatory factor analysis, the DWLS (Diagonally Weighted Least Squares) estimator was used (Table 4).

The confirmatory factor analysis indicates that the proposed factor model is significantly better than the baseline model. Although the Chi-square test reveals a significant mismatch between the model and the data (*p* < 0.001), this result is common for large samples. It is essential to also consider other fit indices (RMSEA, CFI, TLI) for a comprehensive evaluation of the quality of the factor model (Table 5).

All reported fit indices indicate that the model fits the observed data very well. The values of CFI, TLI, NNFI, NFI, RFI, IFI, and RNI are all very close to or above 0.95, indicating excellent fit. While the PNFI is slightly lower, it still suggests a good fit, reflecting a balance between the model’s fit and its parsimony.

The results indicate a moderate to good fit of the model, according to most fit indices. While RMSEA and SRMR suggest a moderate fit, the GFI and CFI values indicate an excellent fit. The McDonald Fit Index (MFI) is relatively low, pointing to a weaker fit based on this specific index. Overall, the model is acceptable but could benefit from improvements to ensure a better fit (Table 6).

All factor loadings are statistically significant (*p* < 0.001), indicating a strong association between each item and its respective factor. High coefficients, especially for I17, I18, I6, and I20, demonstrate that these items contribute significantly to their respective factors. The narrow confidence intervals confirm the precision of the estimates. These findings suggest good construct validity for each measured factor, with significant indicators and high loadings in most cases (Table 7).

Fixing variances at 1.000 is a common practice in CFA models to ensure the interpretability of results. In this case, the values indicate that the model is well-defined. The fact that all variances are equal and fixed suggests that all factors were considered at the same level of importance in the data analysis. Since the variances are fixed, we cannot interpret additional variations or differences between factors based on variance. This approach simplifies interpretation and ensures that factor loadings and correlations between factors remain interpretable without the influence of differing variances among factors (Table 8).

All covariances between the analyzed factors are high and statistically significant, with p-values below 0.001. The confidence intervals are generally narrow, indicating precision in the covariance estimates. The strongest correlations are observed between Factors 2 and 3 (0.811), as well as between Factors 3 and 4 (0.810), suggesting a close relationship between these factors. These data indicate a strong relationship among most factors, which may suggest that these factors measure interrelated or overlapping constructs to some extent (Table 9).

Indicators I17 and I18 have very low residual variances (0.066 and 0.091), indicating that these indicators are very well explained by the common factors. Indicators I3, I10, I5, I6, I7, I19, I8, I12, I14, I23, I24, and I25 exhibit moderate residual variances, suggesting that while they are well explained by the common factors, a significant proportion of their variance remains unexplained. Indicators I4, I11, I26, I21, and I22 have high residual variances, indicating that a substantial portion of their variance is not accounted for by the common factors. These findings suggest that most indicators are well explained by the common factors; however, a few indicators (such as I4, I11, and I26) may require further examination to better understand the sources of their unexplained variance (Table 10).

The table below presents the demographic and obstetric characteristics of a sample of 1550 participants, highlighting variables such as place of origin, education level, occupational status, marital status, ethnicity, previous births, participation in childbirth preparation courses, pregnancy trimester, preferred birth method, and chosen place of birth.

The results are expressed as mean and standard deviation (Mean ± SD) for each analyzed category, and statistical significance is assessed using *t* and *F* tests, with corresponding *p*-values for each comparison (Table 11).

The analysis of Welch’s bidirectional *t*-tests did not reveal statistically significant differences between pregnant women from rural and urban areas regarding their scores for physical environment, psychological well-being, social relationships, and overall environment. However, the mean scores for the psychological domain and environment are higher in urban areas, and the p-values are relatively close to the threshold for significance, suggesting trends that may warrant further exploration in future studies.

In the psychological, social relationships, and environmental domains, the level of education has a significant impact on the scores, suggesting that education plays an important role in these aspects of life. Conversely, in the physical domain, no statistically significant differences were found between groups based on education level, indicating that education does not significantly influence the perceptions or physical condition of participants in this sample. Statistically significant differences were observed among the three education levels. There is a significant psychological difference between the University and Primary School groups, with individuals holding higher education degrees reporting a more favorable psychological perception. The differences between other groups (e.g., “University“ vs. “High school“ and “Primary School“ vs. “High school“) are not statistically significant. These results suggest that a higher level of education is associated with better psychological perception, especially when comparing groups with markedly different education levels (e.g., “University“ vs. “Primary School“). Individuals with higher education have a significantly better perception of social relationships compared to those with secondary education. There are no significant differences between the groups “University” and “High School” or “Primary School” and “High School.” The results indicate that a higher educational level may positively influence the perception of social relationships; however, this effect is not consistent across all educational groups.

The analysis of Welch’s two-tailed t-tests revealed statistically significant differences between employed individuals and those unemployed or with occasional jobs in terms of social relationships and the general environment, with employed individuals scoring higher. For the physical and psychological domains, no statistically significant differences were observed, although the psychological domain had a p-value relatively close to the threshold of significance. These results suggest that employment may have a positive impact on social relationships and the perception of the general environment.

The analysis of Welch’s two-tailed t-tests showed a statistically significant difference between individuals without a partner and those with a partner in terms of social relationships, with individuals with a partner scoring higher. For the physical, psychological, and general environment domains, no statistically significant differences were observed. These results suggest that having a partner may positively impact social relationships.

The analysis of Welch’s two-tailed t-tests showed no statistically significant differences between individuals of other ethnicities and those of Romanian ethnicity regarding scores for the physical environment, psychological environment, social relationships, and general environment. These results suggest that ethnicity does not significantly influence perceptions in these areas.

In all four analyzed dimensions, the differences between women with previous births and those without previous births are not statistically significant. The experience of previous births does not appear to have a measurable impact on perceived quality of life in the physical, psychological, social relationships, or environmental dimensions. These results suggest that other variables (e.g., social support, education, economic status) may play a more significant role in influencing quality of life.

The physical and psychological dimensions are most affected in the third trimester, reflecting increased physical and mental discomfort as the pregnancy progresses. Social relationships remain relatively stable throughout pregnancy, indicating a lesser influence of pregnancy on this aspect. The environmental dimension is perceived more positively in the second trimester, which may coincide with a period of physical and psychological stability before the challenges of the final trimester. These results highlight the importance of physical, psychological, and social support for pregnant women, particularly in the later stages of pregnancy.

Values of *p* > 0.05 for all dimensions indicate that the pregnancy trimester does not significantly influence the quality of life in any evaluated dimension (physical, psychological, social, or environmental). The physical dimension has a p-value of 0.081, close to the significance threshold, suggesting a potential variation that warrants closer investigation. The results indicate that factors other than the pregnancy trimester may play a more significant role in determining the quality of life for pregnant women.

All *p*-values > 0.05, indicating no significant differences between women who participated in prenatal education classes and those who did not for all evaluated dimensions. Although the mean differences suggest trends (e.g., higher scores for participants across all dimensions), these differences are not large enough to be statistically significant. Despite the lack of statistical significance, trends show that pregnant women who participated in prenatal education classes have higher average scores in all domains compared to those who did not participate. This may suggest a real effect, but one that is not strong enough to be detected with statistical significance in this sample.

Women who choose vaginal birth tend to report higher scores in the physical, psychological, and general environment dimensions, suggesting more positive perceptions in these areas. Women who prefer the medical team to decide report higher scores in social relationships, indicating a possible association between delegating the decision and better-perceived social relationships. Women who opt for cesarean delivery have lower scores across all dimensions, which may suggest more negative perceptions compared to the other groups.

The ANOVA analysis for the physical domain shows no statistically significant differences between the groups analyzed based on the mode of delivery. A *p*-value of 0.256 indicates that any observed differences are likely due to chance. These results suggest that, in this sample, the mode of delivery does not significantly influence physical scores.

The *p*-value of 0.085 is higher than the common threshold of 0.05 but relatively close to it. While there is no strict statistical significance (*p* < 0.05), the result suggests that there might be a significant difference between the groups in terms of psychological scores; however, random variability cannot be completely ruled out. The results indicate that the mode of delivery might have an effect on participants’ psychological scores, but not strong enough to be considered statistically significant with this sample and threshold.

The ANOVA analysis for the social relationships domain shows no statistically significant differences between the groups analyzed based on the mode of delivery. A *p*-value of 0.171 indicates that any observed differences are likely due to chance. These results suggest that, in this sample, the mode of delivery does not significantly influence social relationship scores. Births in public hospitals are associated with the lowest average scores across all dimensions (physical, psychological, social relationships, and environment). This may reflect differences in perceptions of service quality, support provided, or conditions in these facilities.

Births in private hospitals and other locations provide more favorable experiences across all dimensions, with a clearer advantage observed in the psychological and physical domains. Births in other locations (possibly home births or specialized centers) are associated with the highest scores in the psychological dimension, suggesting that women may perceive these locations as safer and more emotionally comfortable. Differences between locations are more pronounced in the physical and psychological dimensions but less noticeable in social relationships and the environment. The perception of physical, psychological, social relationships, and general environment is more positive for women who give birth in private hospitals and other locations compared to those who give birth in public hospitals. Women who give birth in other locations report the highest psychological scores, suggesting that these locations might provide a more favorable psychological environment.

## 4. Discussion

In this study, we aimed to evaluate the structural validity, internal consistency, and construct validity of the WHOQOL-BREF scale in a sample of pregnant women. The WHOQOL-BREF questionnaire proved to be a valid and reliable instrument for assessing the quality of life among pregnant women in Romania. The results obtained are comparable to those of other international studies, emphasizing its global applicability [40,41,42,43,44,45,46]. This tool provides a multidimensional perspective on quality of life, enabling the evaluation of physical, psychological, social, and environmental aspects. Validating it in the Romanian context is an important step toward its implementation in future research.

The results highlight a clear relationship between educational and professional status and higher quality-of-life scores, especially in the physical and psychological domains. Women with higher education and stable jobs reported a better perception of their health, which can be explained by greater access to information, resources, and quality medical services. This is consistent with other studies showing that women in urban areas with higher education benefit from improved quality of life due to access to medical and social resources [47,48]. Other research confirms that professional and educational status significantly contributes to a positive perception of health and social relationships, indicating a global trend [49,50]. Studies conducted by the World Health Organization (WHO) underline that higher education is a significant predictor of positive health outcomes, including during pregnancy. Moreover, access to stable jobs provides financial security, reducing the stress associated with pregnancy [51,52].

In our study, an analysis of birth settings revealed that women who choose private hospitals or other locations outside public hospitals report higher scores, particularly in the psychological domain. This result can be attributed to increased comfort, personalized attention, and greater privacy offered by private facilities. A more patient-friendly environment contributes to reducing anxiety and stress associated with childbirth. However, access to private hospitals is influenced by economic factors, limiting the positive impact of these services for women from disadvantaged backgrounds. Other international research supports these conclusions, showing that private hospitals are associated with higher patient satisfaction, particularly due to personalized services and increased care time [53,54].

Although statistical differences between women who participated in prenatal education courses and those who did not were not significant, there is a clear trend toward improved quality-of-life perception among participants. Participation in such programs contributes to increased confidence in the birthing process and reduced anxiety about the unknown. Previous studies have demonstrated that prenatal education is an effective tool for reducing the risk of complications and improving mothers’ psychological adaptation. These programs provide valuable information about pain management, nutrition, and postnatal care, thus contributing to a more positive experience [4,55,56,57].

Regarding results obtained during different trimesters of pregnancy, they indicate a decline in physical and psychological domain scores in the third trimester of pregnancy. This decline can be explained by increased physical discomfort, fatigue, and anxiety related to the approaching childbirth. Social relationships appear to remain constant throughout pregnancy, suggesting stable support from family and the community. These results are consistent with other research emphasizing that the third trimester is a period of increased vulnerability, both physically and psychologically. Women in this stage benefit from additional support from healthcare professionals to cope with challenges [58,59,60].

Additionally, the study highlighted that women with partners report a more positive perception of social relationships, underscoring the importance of family support during pregnancy. The presence of a partner provides emotional stability and practical support, improving quality of life. This aspect is supported by the literature, which shows that adequate social support is a strong predictor of psychological and physical well-being during pregnancy [61,62,63].

## 5. Limitations

The statistical analyses in this study are based exclusively on the information provided and the subjective opinions of the pregnant women included in the sample groups, without any means of verifying the authenticity or accuracy of some responses.

An important limitation of this study is that, being conducted online, there was a tendency for an overrepresentation of participants with higher education and stable employment, which may limit the generalizability of the results to the entire population of pregnant women in Romania.

Another limitation was that the survey was primarily conducted among women who were able to complete it online, and distribution via social media excluded the possibility of direct control over the respondents or calculation of the response rate. Nevertheless, there was no incentive for introducing dishonesty in the responses.

## 6. Conclusions

The study demonstrated that educational, socio-economic factors, and social support significantly influence the quality of life of pregnant women in Romania. The WHOQOL-BREF instrument proved to be valid and reliable for its assessment. Women with higher education and stable employment reported higher scores in the physical and psychological domains, while births in private hospitals or alternative locations were associated with a more positive perception of the childbirth experience. Participation in prenatal courses showed a trend towards improved quality of life, although without statistically significant differences. In the third trimester, scores in the physical and psychological domains declined, while social relationships remained stable. The study emphasizes the need for policies that support prenatal education, equitable access to medical services, and the improvement of public healthcare services.

## Figures and Tables

**Table 1:** Frequentist Scale Reliability Statistics.

Estimate	McDonald’s ω	Cronbach’s α	Average Inter Item Correlation
Point estimate	0.926	0.926	0.333
95% CI lower bound	0.909	0.907	0.269
95% CI upper bound	0.943	0.941	0.390

**Table 2:** Item-Rest Correlation for Dimensions D1–D4.

Item-Rest	Correlation	D1	D2	D3	D4
I1	0.460				
I2	0.410				
I3	0.500	0.729			
I4	0.394	0.521			
I10	0.569	0.664			
I15	0.515	0.547			
I16	0.593	0.570			
I17	0.676	0.806			
I18	0.662	0.757			
I5	0.673		0.628		
I6	0.663		0.688		
I7	0.665		0.611		
I11	0.424		0.518		
I19	0.652		0.657		
I26	0.480		0.458		
I20	0.689			0.635	
I21	0.467			0.419	
I22	0.503			0.472	
I8	0.663				0.639
I9	0.487				0.547
I12	0.620				0.703
I13	0.488				0.588
I14	0.572				0.519
I23	0.555				0.615
I24	0.496				0.574
I25	0.548				0.666

**Table 3:** Estimates of McDonald’s ω, Cronbach’s α, and the average inter-item correlation for dimensions D1–D4.

Estimate	McDonald’s ω				Cronbach’s α				Average Inter Item Correlation			
	D1	D2	D3	D4	D1	D2	D3	D4	D1	D2	D3	D4
Point estimate	0.866	0.819	0.685	0.860	0.873	0.821	0.679	0.857	0.500	0.443	0.441	0.436
95% CI lower bound	0.833	0.774	0.605	0.827	0.839	0.772	0.578	0.820	0.429	0.351	0.304	0.370
95% CI upper bound	0.898	0.864	0.765	0.894	0.901	0.862	0.759	0.889	0.569	0.519	0.569	0.505

**Table 4:** Chi-square test.

Model	Χ²	df	*p*
Baseline model	17475.591	276	
Factor model	497.335	246	<0.001

**Table 5:** Fit indices.

Index	Value
Comparative Fit Index (CFI)	0.985
Tucker-Lewis Index (TLI)	0.984
Bentler-Bonett Non-normed Fit Index (NNFI)	0.984
Bentler-Bonett Normed Fit Index (NFI)	0.972
Parsimony Normed Fit Index (PNFI)	0.866
Bollen’s Relative Fit Index (RFI)	0.968
Bollen’s Incremental Fit Index (IFI)	0.985
Relative Noncentrality Index (RNI)	0.985

**Table 6:** Other fit measures.

Metric	Value
Root mean square error of approximation (RMSEA)	0.082
RMSEA 90% CI lower bound	0.071
RMSEA 90% CI upper bound	0.092
RMSEA *p*-value	1.081 × 10^−6^
Standardized root mean square residual (SRMR)	0.087
Hoelter’s critical N (α = 0.05)	88.242
Hoelter’s critical N (α = 0.01)	93.452
Goodness of fit index (GFI)	0.978
McDonald’s fit index (MFI)	0.440
Expected cross-validation index (ECVI)	

**Table 7:** Factor loadings.

					95% Confidence Interval		
Factor	Indicator	Estimate	Std. Error	z-Value	*p*	Lower	Upper
Factor 1	I3	0.727	0.024	30.807	<0.001	0.681	0.774
	I4	0.599	0.029	20.996	<0.001	0.543	0.655
	I10	0.760	0.023	32.662	<0.001	0.714	0.805
	I15	0.672	0.025	27.378	<0.001	0.624	0.720
	I16	0.727	0.023	31.376	<0.001	0.682	0.773
	I17	0.967	0.019	51.664	<0.001	0.930	1.003
	I18	0.953	0.019	50.777	<0.001	0.916	0.990
Factor 2	I5	0.817	0.022	36.330	<0.001	0.773	0.862
	I6	0.899	0.024	37.650	<0.001	0.852	0.945
	I7	0.791	0.024	33.122	<0.001	0.744	0.838
	I11	0.568	0.025	22.942	<0.001	0.520	0.617
	I19	0.806	0.024	33.448	<0.001	0.759	0.853
	I26	0.607	0.029	20.938	<0.001	0.550	0.664
Factor 3	I20	0.908	0.034	26.464	<0.001	0.841	0.976
	I21	0.604	0.030	20.363	<0.001	0.546	0.662
	I22	0.657	0.027	24.253	<0.001	0.604	0.710
Factor 4	I8	0.820	0.024	34.631	<0.001	0.773	0.866
	I9	0.651	0.023	27.904	<0.001	0.606	0.697
	I12	0.783	0.023	34.772	<0.001	0.739	0.827
	I13	0.695	0.023	30.622	<0.001	0.651	0.740
	I14	0.733	0.022	33.292	<0.001	0.689	0.776
	I23	0.748	0.025	29.416	<0.001	0.698	0.798
	I24	0.694	0.023	29.722	<0.001	0.648	0.739
	I25	0.739	0.023	31.483	<0.001	0.693	0.785

**Table 8:** Factor variances.

					95% Confidence Interval	
Factor	Estimate	Std. Error	z-Value	*p*	Lower	Upper
Factor 1	1.000	0.000			1.000	1.000
Factor 2	1.000	0.000			1.000	1.000
Factor 3	1.000	0.000			1.000	1.000
Factor 4	1.000	0.000			1.000	1.000

**Table 9:** Factor covariances.

							95% Confidence Interval	
			Estimate	Std. Error	z-Value	*p*	Lower	Upper
Factor 1	↔	Factor 2	0.714	0.022	33.124	<0.001	0.672	0.756
Factor 1	↔	Factor 3	0.593	0.032	18.665	<0.001	0.531	0.655
Factor 1	↔	Factor 4	0.537	0.019	28.950	<0.001	0.500	0.573
Factor 2	↔	Factor 3	0.811	0.039	20.549	<0.001	0.734	0.888
Factor 2	↔	Factor 4	0.769	0.022	34.567	<0.001	0.725	0.812
Factor 3	↔	Factor 4	0.810	0.036	22.308	<0.001	0.739	0.881

**Table 10:** Residual variances.

			95% Confidence Interval	
Indicator	Estimate	Std. Error	Lower	Upper
I3	0.471	0.000	0.471	0.471
I4	0.642	0.000	0.642	0.642
I10	0.423	0.000	0.423	0.423
I15	0.548	0.000	0.548	0.548
I16	0.471	0.000	0.471	0.471
I17	0.066	0.000	0.066	0.066
I18	0.091	0.000	0.091	0.091
I5	0.332	0.000	0.332	0.332
I6	0.193	0.000	0.193	0.193
I7	0.374	0.000	0.374	0.374
I11	0.677	0.000	0.677	0.677
I19	0.350	0.000	0.350	0.350
I26	0.632	0.000	0.632	0.632
I20	0.175	0.000	0.175	0.175
I21	0.636	0.000	0.636	0.636
I22	0.568	0.000	0.568	0.568
I8	0.328	0.000	0.328	0.328
I9	0.576	0.000	0.576	0.576
I12	0.387	0.000	0.387	0.387
I13	0.517	0.000	0.517	0.517
I14	0.463	0.000	0.463	0.463
I23	0.441	0.000	0.441	0.441
I24	0.519	0.000	0.519	0.519
I25	0.454	0.000	0.454	0.454

**Table 11:** Distribution of physical, psychological, social and environmental quality of life domains by demographic data (n = 1550).

Demographic and Obstetric Characteristics		N (%)	Mean ± SD			
		n = 1550	D1	D2	D3	D4
Place of origin	rural	460 (29.67%)	63.35 ± 19.87	72.37 ± 15.82	67.21 ± 22.25	72.55 ± 18.80
	urban	1090 (70.32%)	67.69 ± 17.99	73.148 17.216	73.14 ± 17.21	78.27 ± 15.15
*p* value			0.206	0.096	0.111	0.072
t			−1.275	−1.684	−1.616	−1.824
Education Level	University	1270 (81.93%)	67.71 ± 18.67	77.44 ± 14.32	73.28 ± 18.05	79.06 ± 15.33
	Primary School	101 (6.51%)	58.73 ± 13.61	61.57 ± 19.74	60.18 ± 20.74	60.76 ± 19.64
	High school	179 (11.54%)	61.27 ± 19.27	69.95 ± 14.06	64.03 ± 21.52	67.43 ± 67.43
*p* value			0.166	0.002 ***	0.026 ***	< 0.001
F			1.817	6.555	3.749	9.451
Occupational status	Unemployed/ Occasional Job	310 (20%)	63.47 ± 16.07	71.23 ± 15.75	63.17 ± 20.04	66.63 ± 17.88
	Employed	1240 (80%)	67.13 ± 19.19	76.69 ± 14.86	73.44 ± 18.21	79.06 ± 15.17
*p* value			0.283	0.088	0.013 ***	< 0.001
t			−1.085	−1.743	−2.595	−3.560
Marital Status	Without partner	380 (24.51%)	64.88 ± 21.43	73.14 ± 13.19	61.57 ± 13.14	71.35 ± 13.77
	With partner	1170 (75.48%)	66.59 ± 18.29	75.91 ± 15.40	72.67 ± 19.29	77.25 ± 16.72
*p* value			0.749	0.420	0.004 ***	0.110
t			−0.820	−3.160	−1.661	−1.661
Ethnicity	Other ethnicities	51 (3.29%)	53.57 ± 18.89	68.45 ± 11.75	73.81 ± 26.10	68.75 ± 21.19
	Romanian	1499 (96.70%)	67.00 ± 18.44	75.93 ± 15.24	71.25 ± 18.69	76.93 ± 16.21
*p* value			0.111	0.149	0.806	0.350
t			−1.840	−1.621	0.255	−1.008
Previous Births	Yes	552 (35.61%)	66.96 ± 19.08	75.40 ± 16.89	70.83 ± 18.99	74.03 ± 16.84
	No	998 (64.38%)	66.10 ± 18.46	75.69 ± 14.26	71.65 ± 19.06	77.84 ± 16.21
*p* value			0.790	0.915	0.801	0.182
t			0.267	−0.107	−0.252	−1.345
Pregnant women’s participation in childbirth preparation	Yes	510 (32.90%)	69.78 ± 16.97	77.58 ± 16.05	74.66 ± 16.66	78.56 ± 15.17
	No	1040 (67.09%)	64.76 ± 19.22	74.63 ± 14.67	69.79 ± 19.88	75.60 ± 17.05
*p* value			0.103	0.276	0.114	0.279
t			1.645	1.095	1.594	1.088
Pregnancy trimester of the pregnant woman	Trimester I	192 (12.38%)	70.83 ± 16.02	76.94 ± 14.00	72.22 ± 19.12	74.68 ± 17.58
	Trimester II	228 (14.70%)	69.30 ± 18.75	77.48 ± 14.63	72.81 ± 19.48	79.46 ± 14.77
	Trimester III	1130 (72.90%)	63.28 ± 19.05	74.13 ± 15.81	70.32 ± 18.84	75.76 ± 16.88
*p* value			0.081	0.441	0.761	0.392
F			2.561	0.822	0.274	0.942
Chosen birth method for the current pregnancy	Cesarean section	481 (31.03%)	62.72 ± 19.09	71.87 ± 16.56	67.53 ± 20.21	74.54 ± 16.63
	I prefer the medical team to decide	205 (13.22%)	67.51 ± 16.44	74.80 ± 16.32	76.19 ± 15.43	76.33 ± 12.63
	Vaginal birth	864 (55.74%)	68.19 ± 18.75	77.89 ± 13.69	72.35 ± 18.86	77.75 ± 17.25
*p*-value			0.256	0.085	0.171	0.560
F			1.375	2.501	1.789	0.582
Chosen place for birth	Another location	160 (10.32%)	69.76 ± 19.83	81.11 ± 10.19	72.77 ± 13.89	77.50 ± 12.62
	Private hospital	398 (25.67%)	70.20 ± 17.20	78.28 ± 14.24	72.80 ± 17.72	77.96 ± 14.90
	Public hospital	992 (64%)	64.46 ± 18.83	73.76 ± 15.83	70.62 ± 20.15	75.89 ± 17.59
*p*-value			0.206	0.096	0.799	0.786
F			1.598	2.376	0.225	0.241

*** *p*-value 0.005.

## Data Availability

The raw data supporting the conclusions of this article will be made available by the authors upon request.

## Public Involvement Statement

No public involvement in any aspect of this research.

## Guidelines and Standards Statement

A complete list of reporting guidelines can be accessed via the equator network: https://www.equator-network.org/ (accessed on 12 December 2024).
